# KbvR mutant of *Klebsiella pneumoniae* affects the synthesis of type 1 fimbriae and provides protection to mice as a live attenuated vaccine

**DOI:** 10.1186/s13567-022-01116-y

**Published:** 2022-11-26

**Authors:** Fusheng Zhang, Yan Meng, Li Xu, Yujiao Tian, Huigai Lu, Jichen Xie, Renhui Ma, Moran Li, Bei Li

**Affiliations:** 1 School of Basic Medicine, Hubei University of Medicine, Shiyan, 442000 Hubei China; 2 School of Biomedical Engineering, Hubei University of Medicine, Shiyan, 442000 Hubei China; 3 Biomedical Research Institute, Hubei University of Medicine, Shiyan, 442000 Hubei China

**Keywords:** *Klebsiella pneumoniae*, KbvR, vaccine, immune response

## Abstract

*Klebsiella pneumoniae* is a leading cause of severe infections in humans and animals, and the emergence of multidrug-resistant strains highlights the need to develop effective vaccines for preventing such infections. Live attenuated vaccines are attractive vaccine candidates available in the veterinary field. We recently characterized that the *K. pneumoniae kbvR* (*Klebsiella* biofilm and virulence regulator) mutant was a highly attenuated strain in the mice model. In the present study, the characterization, safety, and protective efficacy of Δ*kbvR* strain as a live attenuated vaccine were evaluated. The synthesis and activity of type 1 fimbriae were increased in the Δ*kbvR* strain. All mice inoculated by the subcutaneous route with 10^5^, 10^6^, and 10^7^ colony-forming units (CFU) doses of the Δ*kbvR* strain survived. Subcutaneous immunization with two doses of 10^5^ or 10^7^ CFU Δ*kbvR* elicited a robust humoral immune response, and provided protection against the following *K. pneumoniae* intraperitoneal infection. The antisera of mice immunized with 10^5^ CFU dose improved the opsonophagocytic ability and complement-mediated lysis not only to the same serotype strain but also to the different serotype strain. The passive transfer of antisera from 10^5^ CFU dose-immunized mice provided protection against *K. pneumoniae* infection. Overall, our results suggest the great potential of the Δ*kbvR* strain as a novel vaccine candidate against *K. pneumoniae* infections in herds or humans.

## Introduction

*Klebsiella pneumoniae* is a ubiquitous bacterium that can be found in a wide range of ecological niches including water, soil, vegetation, and insects [1–3]. It also can be recovered from the stools, nasopharyngeal swabs, or urine samples collected from companion and farm animals, such as dogs, cats, horses, cows, chickens, and pigs. To these animals, it is an essential cause of pneumonia, metritis, septicemia, and mastitis [4–6]. The infection leads to losses in milk production, food contamination, and high mortalities [7–9]. Meanwhile, the *K. pneumoniae* in animal hosts and along the food chain can be transmitted to humans and cause infection [10–12]. Treatment with antibiotics is an important method against bacterial pathogens. However, more than one-third of *K. pneumoniae* isolates in Europe are resistant to at least one class of the antimicrobial agents, and multidrug-resistant (MDR) strains have been recovered in clinical isolates not only from humans but also from companion animals and livestock [13–15].

Given the increasing multidrug resistance, new strategies are needed to control and prevent infection by MDR *K. pneumoniae* in animals and humans. Vaccination is regarded as another effective method to prevent infectious diseases. To date, there are no vaccines available against *K. pneumoniae* infection and the development of vaccines against *K. pneumoniae* has become a priority in many countries. The schemes for vaccine development against *K. pneumoniae* include whole cell vaccines, ribosomal vaccines, outer membrane vesicles, and virulence factors including capsular polysaccharides (CPS), lipopolysaccharide, and protein-based vaccines [16–21]. Live attenuated vaccines are a mode of whole cell vaccines that are more immunogenic and can elicit long-lasting immune responses than the subunit vaccines, and they have a relatively low production cost. Although the problem of biological safety limits the use of live vaccines, they are suitable to immunizing large communities or herds. The main vaccines used for animals or humans against the infection of *Brucella melitensis*, *Yersinia pestis*, and *Mycobacterium tuberculosis* are still live attenuated vaccines [22–26]. For *K. pneumoniae*, a *tonB* deletion mutant strain can induce a protective immune response against challenge with a wild-type strain [27].

KbvR is a regulator that has been found to reduce biofilm formation and the synthesis of CPS in the *K. pneumoniae* NTUH-K2044 strain. The virulence of the *kbvR* mutant was significantly decreased in the murine model by intraperitoneal injection [28]. RNA-seq results show that the expression levels of type 1 fimbriae genes such as *fimA* and *fimH* were increased in *K. pneumoniae kbvR* mutant strain [28]. Fimbriae are filamentous structures present on the cell surface of bacteria. The components of fimbriae have been shown to be promising vaccine candidates [29, 30]. The selected T- and B-cell specific epitopes of type 1 fimbriae FimA, FimF, FimG, and FimH proteins have been estimated to be used as potential vaccine candidates against *K. pneumoniae* infection, and six epitopes confirmed by molecular dynamics can be applied for vaccine development [31, 32]. Thus, the Δ*kbvR* strain might be a potentially viable option for the preparation of vaccines in preventing *K. pneumoniae* infections in humans and animals. In this study, the influence of KbvR on the synthesis of type I fimbriae was investigated. Then, experiments on the safety, antibody test, and protective efficacy of the Δ*kbvR* strain in mice were performed to evaluate the potential of the *kbvR* mutant strain as a live attenuated vaccine.

## Materials and methods

### Bacterial strains and growth conditions

The capsular serotype K1 hypervirulent *K. pneumoniae* strain NTUH-K2044 (WT), the NTUH-K2044 *kbvR* gene deletion strain (Δ*kbvR*), and the capsular serotype K3 standard *K. pneumoniae* strain ATCC 13,883 were preserved in our laboratory. All strains from frozen stocks were inoculated into Luria–Bertani (LB) broth and grown overnight with shaking at 37 °C. Then, the cultures were diluted 1:100 into fresh LB media the following day and cultured to the desired phase of growth.

### RNA isolation and RT-PCR

Total RNA were extracted from mid-log phase *K. pneumoniae* WT and Δ*kbvR* strains using an RNeasyMidi kit (Qiagen). Purified RNA was DNase-treated with RNase-free DNase I (Qiagen) to eliminate the residual DNA. The quality and quantity of RNA were determined via 1% agarose gel electrophoresis and with a Nano Drop 2000 UV–Vis Spectrophotometer (Thermo Scientific). The first-strand cDNA synthesis was conducted using the first-strand cDNA synthesis kit (Promega) and quantified. The relative expression levels of *fimA* and *fimH* genes in WT and Δ*kbvR* strains were analyzed by agarose gel electrophoresis after amplifying cDNA by PCR. 16 S rRNA was used as the normalized gene. The relative mRNA levels were quantified by densitometry using ImageJ software and then calculated by dividing the densitometry of *fimA*/*fimH* gene by the corresponding densitometry of 16 S rRNA.

### Yeast agglutination assay

The presence and activity of type I fimbriae at the bacterial cell surface were assessed using the baker’s yeast suspended in phosphate-buffered saline (PBS) as previously described [33]. The mid-log phase of statically cultured WT and Δ*kbvR* strains were washed twice and resuspended in PBS with 10^8^ colony-forming units (CFU)/mL. Equal cell number of 50 µL yeast cell suspension and 50 µL different bacterial suspensions were mixed on a glass slide. After shaking at room temperature for 3 min, the aggregation was assessed by visual inspection.

### Transmission electron microscopy

The WT or Δ*kbvR* strain was statically cultured in LB broth for 10 h and suspended in 1 mL of PBS for negative staining to observe the fimbriae directly [34]. A glow-discharged formvar-coated copper grid was floated on a drop of each bacterial suspension liquid for 3 min. Then, the excess liquid was drained off with filter paper, and the copper grid was stained with uranyl acetate for another 3 min followed by air-drying. The specimens were viewed under a transmission electron microscope (HT7800, Japan) operated at an accelerating voltage of 120 kV.

### Mouse subcutaneous infections

Five- to six-week-old female BALB/c mice were obtained from HUNAN SJA laboratory animal Co., Ltd. to determine the virulence of Δ*kbvR* in mice by subcutaneous route. The NTUH-K2044 WT and Δ*kbvR* strains were cultured to OD_600_ = 1.2 in LB broth and serially 10-fold diluted with sterile PBS. Appropriate dilutions were plated onto LB agar plates to calculate the numbers of CFU. A total of 10 mice for each group gathered from two experiments were injected subcutaneously with 10^5^, 10^6^ CFU WT or 10^5^, 10^6^, 10^7^ CFU Δ*kbvR*. The mice were monitored daily for 14 days, and the survival rates were calculated to analyze the virulence of Δ*kbvR* by subcutaneous route.

### Mouse immunizations and challenge experiments

Two different groups of BALB/c mice were respectively immunized with a low dose (10^5^ CFU) or high dose (10^7^ CFU) Δ*kbvR* strain in 100 µL PBS two times for 4 weeks at 2-week intervals by subcutaneous route. The mice immunized with PBS were used as a normal control. All mice were monitored for skin reaction and weighed daily. At 28 days after the first immunization, the serum samples were collected from immunized (*n* = 10 for 10^5^ CFU dose from two experiments and *n* = 5 for 10^7^ CFU dose) and normal control (*n* = 10 from two experiments) mice to evaluate antibody production by enzyme-linked immunosorbent assay (ELISA). The liver, spleen, and lungs of the mice were processed, paraffin-embedded, microtome sectioned, and stained with hematoxylin and eosin to determine histopathological changes using an Olympus DP74 camera.

Two weeks after the second immunization, the mice (10^5^ CFU Δ*kbvR*-immunized group and PBS control group) were infected with 10^4^ or 10^5^ CFU log-phase *K. pneumoniae* WT strain via intraperitoneal route. At the 12 h post-infection, five immunized or control mice challenged by 10^5^ CFU WT strain were sacrificed. The blood was collected and plated onto LB plate to calculate the number of bacteria. Then, 3 mL LB medium was injected into the abdominal cavity of the mice. A total of 10 µL peritoneal lavage fluid was collected and serially diluted to calculate the bacterial number, while another 10 µL peritoneal lavage fluid was coated onto the slides and stained via the gram stain method to be observed by optical microscopy. Thereafter, the liver, spleen, and lungs were removed aseptically, weighed, and homogenized in PBS using a tissue homogenizer. The samples were serially diluted and spread on LB agar plate containing 100 µg/mL ampicillin to determine the bacterial counts. The remaining mice challenged by 10^4^ or 10^5^ CFU WT (*n* = 7 for each PBS control group and *n* = 8 for each immunized group) were observed every day for the next 14 days to evaluate the survival rate.

### Enzyme-linked immunosorbent assay

Relative antibody levels in mice were determined by ELISA [35]. First, 96-well flat-bottom plates were coated with 100 µL lysate of whole NTUH-K2044 WT cells. This WT strain was grown in LB broth to OD_600_ = 1.2, collected by centrifugation, resuspended in PBS, and lysed by sonication. After overnight incubation at 4 °C, plates were washed three times with PBS including 0.05% Tween-20 (PBST), blocked with 5% nonfat dry milk for 1 h, and washed three times with PBST. The serum samples of immunized or normal control mice were diluted 1:400. The resulting titer was corrected at an optical density of 450 nm to about 0.1 of control serum, and 100 µL was applied per well followed by 1.5 h incubation at 37 °C. After washing with PBST, 100 µL of 1:3000 horseradish peroxidase-conjugated goat anti-mouse immunoglobulin G (IgG) antibodies was added and incubated at 37 °C for 1 h. The color was developed for 10 min in the dark by addition of 100 µL 3, 30, 5, 50-tetramethylbenzidine, and the reaction was stopped by adding 100 µL 2 M H_2_SO_4_. The optical density of the reaction was recorded at 450 nm using an ELISA plate reader and corrected by subtracting the optical density of a blank control.

### Opsonophagocytic assay

For an opsonophagocytic assay, plasmid pLac-EGFP with a gene encoding green fluorescence protein was electroporated into *K. pneumoniae* NTUH-K2044 and ATCC 13,883 strains. The bacteria were cultured in LB at 37 °C to OD_600_ = 1.2, washed with PBS, and adjusted to approximately 10^7^ CFU/mL in RPMI 1640 medium. RAW264.7 macrophage cells were seeded in 24-well glass bottom culture plates and cultured in RPMI 1640 medium containing 2 mM glutamine and 10% (v/v) fetal calf serum [36]. For the opsonophagocytic assay, macrophages were infected with bacteria (NTUH-K2044 or ATCC 13,883) at a multiplicity of infection (MOI) of 50 bacteria/macrophage and 1 µL pooled immunized or normal mice sera (*n* = 5 for each group) in a 1100 µL reaction at 37 °C. After 120 min of contact, cells were washed twice with PBS and incubated for an additional 90 min with RPMI 1640 medium in the presence of 500 µg/mL gentamicin to eliminate the remaining extracellular bacteria. Then, the cells were washed, fixed, and stained with Alexa Fluor 594 dye (red) for actin cytoskeleton and DAPI (blue) for host cell nuclei. Finally, the cells were observed by confocal microscopy. For the opsonophagocytic activity against NTUH-K2044, the sum of the intracellular bacteria in the macrophages in different fields were calculated and quantified within 300 cells. For the opsonophagocytic activity against ATCC 13,883, the washed cells were lysed with 0.5% Triton X-100 and serial dilutions were plated on LB agar to quantify intracellular bacteria. The mean opsonophagocytic ratio of sera to ATCC 13,883 was determined using the formula = 100 ⋅ CFU of intracellular phagocytized bacteria/CFU of input bacteria. Experiments were conducted on three independent occasions [36].

### Complement-mediated antibacterial test

The complement-mediated antibacterial test was performed as previously described [36, 37]. The pooled immunized or normal mice sera (*n* = 5) were heat-inactivated in a 56 ℃ water bath for 1 h. A total of 10^6^ bacterial cells (*K. pneumoniae* strain NTUH-K2044 or ATCC 13,883) were co-incubated with 25% (v/v) human serum (providing the complement) donated by healthy volunteers with or without 5 µL heat-inactivated mice sera in a 50 µL reaction at 37 ℃ for 2 h. The colony count was calculated by the serial dilution method. The mean survival ratio was determined using the formula = 100 ⋅ CFU of experimental wells with mice sera/CFU of control wells without mice sera.

### Mouse passive immunization and challenge assay

For passive immunization experiments, the sera from 5 mice immunized with two doses of 10^5^ CFU *K. pneumoniae* Δ*kbvR* strain (immunized sera) or from 5 mice injected with PBS (normal sera) were collected and pooled respectively. Then, pooled sera from immunized or normal mice were passively transferred to groups of 5 naïve female BALB/c mice in the dose of 1 µL respectively along with 100 µL 10^4^ CFU WT by intraperitoneal route. Mice were observed daily for 14 days, and survival was monitored [21].

### Statistical analyses

The data were presented either as mean ± standard error (SD). A Mantel–Cox (log-rank) test was used to compare the survival curves, and the Student *t*-test was used for the statistical comparisons between the two groups.

## Results

### Type 1 fimbriae biosynthesis is increased in *kbvR* mutant strain

**Figure 1:**
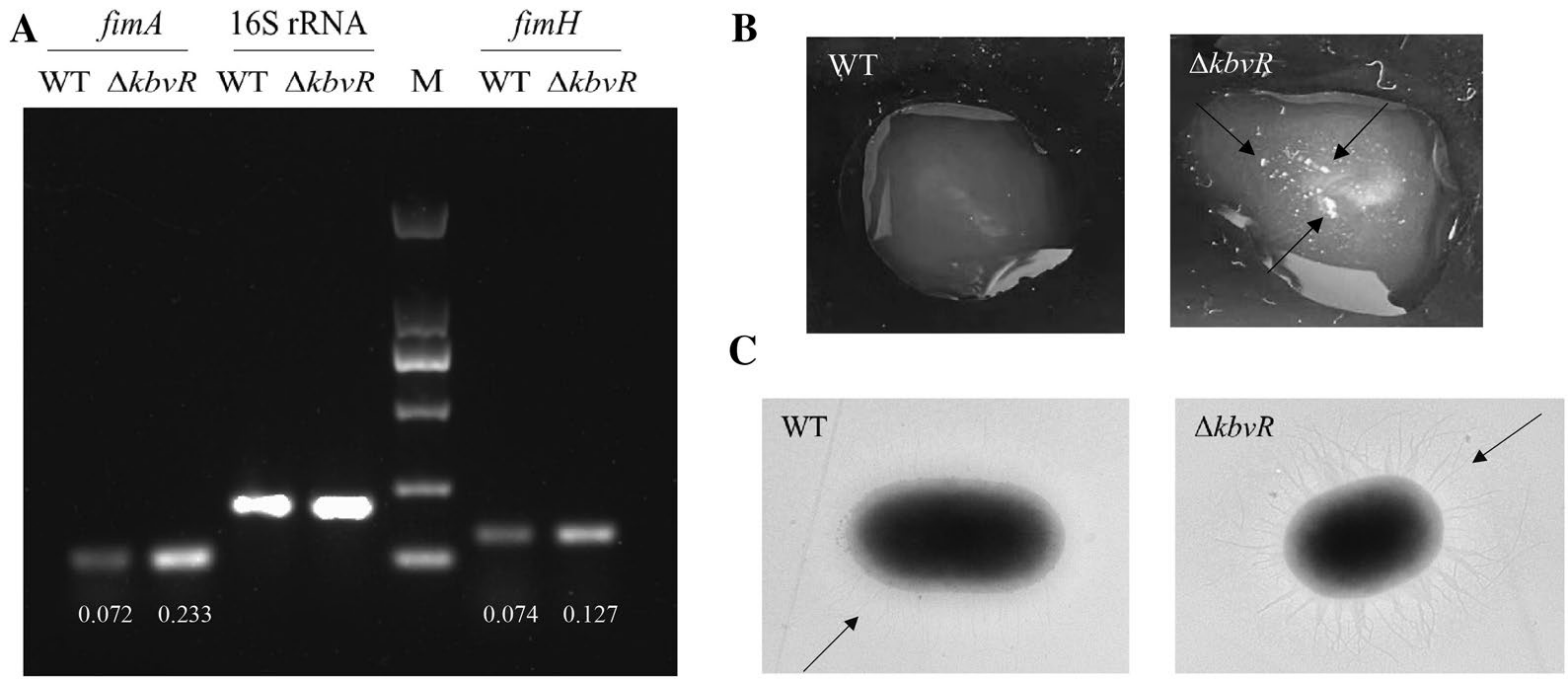
**KbvR negatively regulates the expression of type I fimbriae. A** mRNA levels of *fimA* and *fimH* genes were detected by RT-PCR. The overnight cultures of WT and Δ*kbvR* strains were diluted 1:100 into fresh LB media and cultured to mid-log phase at 37 °C. Total RNA were extracted and RT-PCR was conducted with 16 S rRNA as the normalized gene. The relative mRNA levels of *fimA* and *fimH* genes were analyzed by agarose gel electrophoresis, quantified by densitometry using ImageJ software, and calculated by dividing the densitometry of *fimA*/*fimH* gene by the corresponding densitometry of 16 S rRNA. **B** Yeast agglutination assay. The mid-log phase WT and Δ*kbvR* cultures (10^8^ CFU/mL, 50 µL) were mixed with equal cell numbers of 50 µL yeast cell suspension and shaken at room temperature for 3 min. Large clumps (with arrows indicated) were observed in the Δ*kbvR* strain compared with those in WT. **C** Transmission electron microscopy images of *K. pneumoniae* NTUH-K2044 and its Δ*kbvR* mutant, and arrows indicate the fimbriae.

In our previous study, the RNA-seq results show that the transcription levels of gene cluster associated with type I fimbriae synthesis were increased in the Δ*kbvR* strain (NCBI GenBank: No. SAMN17192750-SAMN17192755) [28]. The RT-PCR confirmed that the transcription levels of *fimA* and *fimH*, the genes that encode the major subunit protein and the adhesion of type I fimbriae, respectively, were increased in the *kbvR* mutant strain. The relative mRNA levels of *fimA* and *fimH* in Δ*kbvR* and WT were 0.233 versus 0.072 and 0.127 versus 0.074, respectively (Figure 1A). The yeast agglutination assay and transmission electron microscopy were used to directly observe the activity and production of type I fimbriae. As shown in Figure 1B, the Δ*kbvR* strain gave obvious clumps in a clear solution, whereas little agglutination was observed for WT at the same number of cells, indicating more type I fimbriae on the surface of Δ*kbvR* than on that of WT, which mediated stronger yeast agglutination. The transmission electron microscopy images also show the existence of a large number of fimbriae on the surface of Δ*kbvR* compared with that of WT (Figure 1C). These results suggest that KbvR negatively regulated the synthesis and activity of type I fimbriae.

### Δ*kbvR* strain injected subcutaneously is avirulent in the mouse model

**Figure 2:**
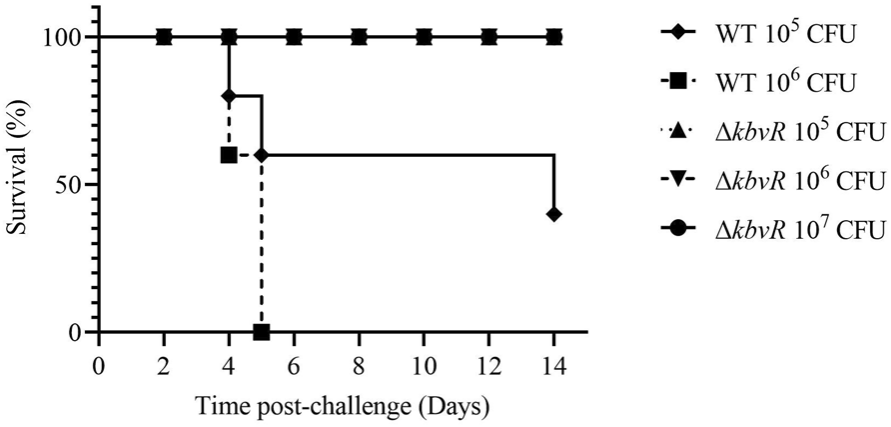
**Survival curves for the** ***K. pneumoniae*** **WT and Δ*kbvR*** **strains by subcutaneous infection**. A total of 5 groups of 10 five- to six-week-old female BALB/c mice were injected subcutaneously with 10^5^, 10^6^ CFU WT or 10^5^, 10^6^, 10^7^ CFU Δ*kbvR*, and the survival rate was measured for 14 days (*P* < 0.0001).

The 50% lethal dose (LD_50_) was equal to 5 × 10^2^ CFU for WT and more than 5 × 10^5^ CFU for Δ*kbvR* by intraperitoneal infection [28]. To assess the virulence of the Δ*kbvR* strain infected by the subcutaneous route, the possible immunization route of live vaccine, the survival rate of mouse model infected subcutaneously by 10^5^, 10^6^ CFU WT and 10^5^, 10^6^, 10^7^ CFU *kbvR* mutant strains were calculated for 14 days. The Δ*kbvR* strain was also highly attenuated via the subcutaneous route. All mice infected by 10^6^ CFU WT died in 5 days, and the survival rate was decreased to 40% in the mice infected by 10^5^ CFU WT. On the contrary, no deaths occurred in the Δ*kbvR*-infected groups which were even challenged by the 10^7^ CFU dose (Figure 2).

### Subcutaneous immunization with *kbvR* strain stimulates humoral immune response

**Figure 3:**
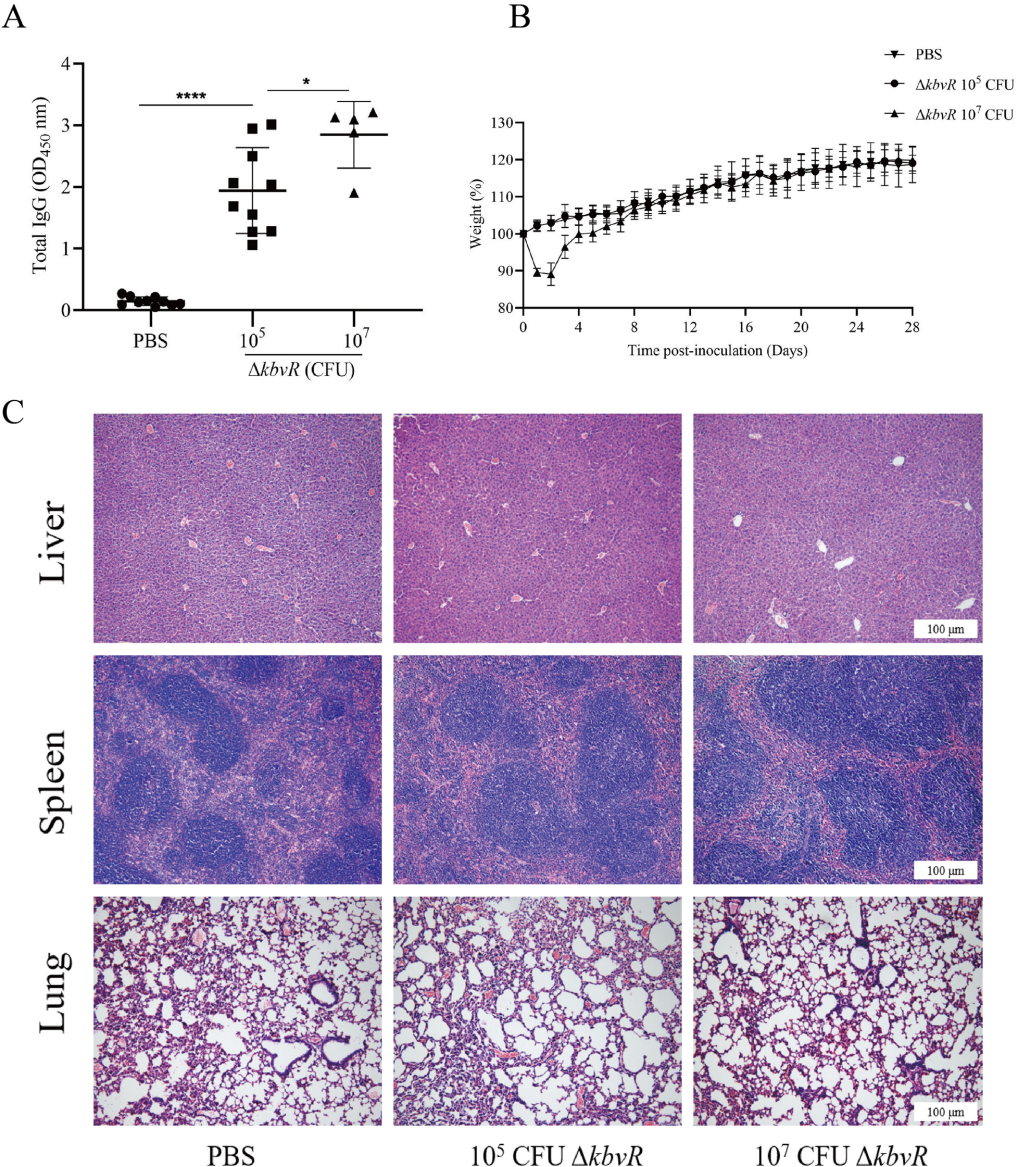
**Immune responses to inoculation with 10 CFU or 10**^**7**^ **CFU Δ*kbvR***. Mice were immunized with 10^5^ CFU (*n* = 10), 10^7^ CFU (*n* = 5) Δ*kbvR* or PBS (*n* = 10) by the subcutaneous route for two doses on days 0 and 14. **A** Blood was collected on day 28 post-inoculation, and the relative levels of serum IgG antibody against whole cell lysates of NTUH-K2044 were determined by ELISA. The optical density of the horseradish peroxidase reaction was recorded at 450 nm. **B** Daily weights of mice inoculated with 10^5^ CFU Δ*kbvR*, 10^7^ CFU Δ*kbvR*, or PBS over 28 days. **C** Histopathological examination of the liver, spleen, and lung of 10^5^, 10^7^ Δ*kbvR* or PBS immunized mice on day 28 post-inoculation. The section was counterstained with hematoxylin and eosin. Scale bars = 100 μm. *: *P* < 0.05; ****: *P* < 0.0001.

Given that Δ*kbvR* could be a live attenuated vaccine candidate, the concentration of IgG in the mice immunized with two doses of 10^5^ or 10^7^ CFU Δ*kbvR* at 2-week intervals were quantified by ELISA assay. All immunized mice mounted IgG antibody response to whole *K. pneumoniae* lysates at day 28 post-immunization, and the concentration was significantly higher than that of the PBS control group (*P* < 0.0001). The results indicate a positive effect of the Δ*kbvR* strain on antibody induction. The 10^7^ CFU-immunized group had even higher antibody concentration than the 10^5^ CFU-immunized group (*P* < 0.05), suggesting that the increase in the immunized Δ*kbvR* dose might improve vaccine efficacy (Figure 3A).

The weights and the skin changes in the injection sites of mice throughout the immunization were measured as indicators of side effects. No skin changes at the sites of injection and weight loss were observed in all mice inoculated with 10^5^ CFU Δ*kbvR*. However, mice initially inoculated with 10^7^ CFU Δ*kbvR* had significantly lower weights (about 12%) than PBS-inoculated controls from days 1–6 post the first inoculation (*P* < 0.05), and local adverse reactions such as skin lesion and a local hair loss at the injection site were observed (Figure 3B). These differences were significant when analyzed by mixed ANOVA study. The mice recovered their body weight on day 8, and no significant weight differences from PBS control were observed after the second administration. The liver, spleen, and lungs of PBS control, 10^5^, or 10^7^ CFU Δ*kbvR*-inoculated mice were examined histologically at 28 days after the first injection to further investigate the virulence and safety of Δ*kbvR* in mice. No histological lesions and inflammatory infiltrate were observed in any examined organ (Figure 3C). These data demonstrate that, although the 10^7^ CFU-immunized dose induced higher humoral response, it also might have the local side effect. A dose of 10^5^ CFU Δ*kbvR* which was well within the range of safe and immunogenic doses was chosen for further evaluation.

### Δ*kbvR* immunization induces protection against *K*. *pneumoniae* intraperitoneal challenge

**Figure 4:**
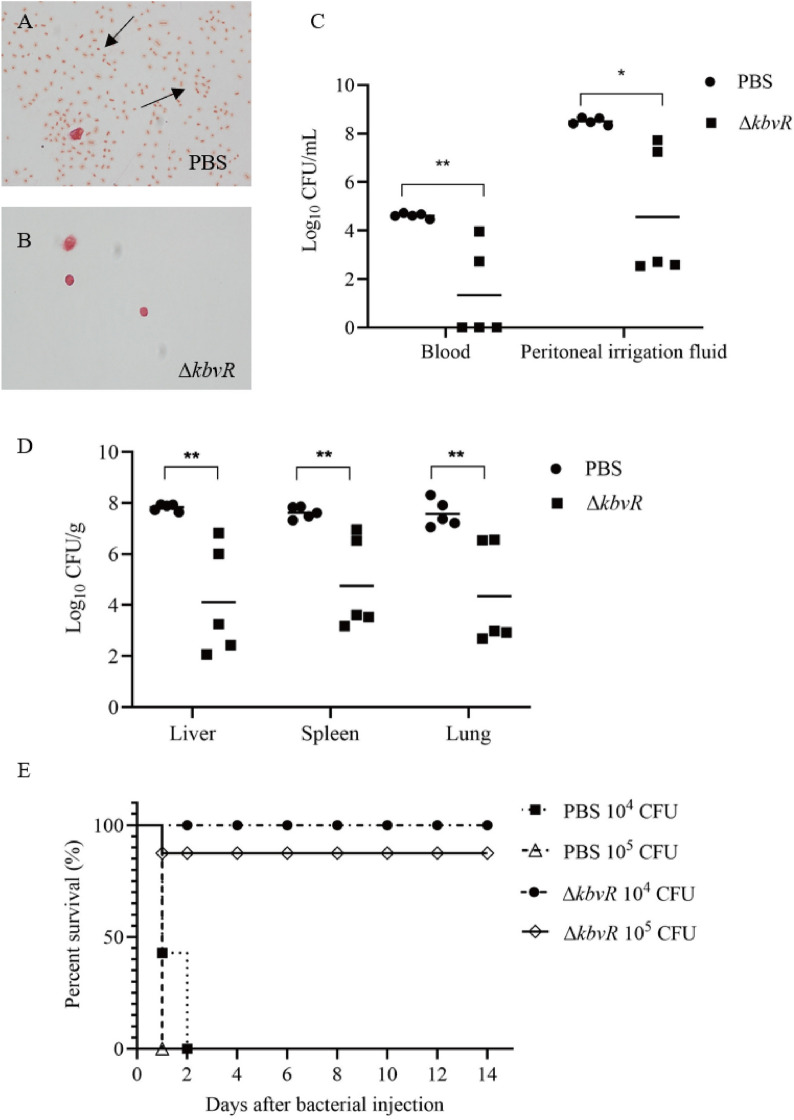
**Δ** ***kbvR*** **immunization induces protection against** ***K. pneumoniae*** **intraperitoneal challenge.** Two doses of 10^5^ CFU Δ*kbvR* strain immunized mice (*n* = 21) or PBS (*n* = 19) inoculated normal mice were intraperitoneally infected with 10^4^ (*n* = 15) or 10^5^ (*n* = 25) CFU log-phase wild-type strain 2 weeks after the second immunization. **A, B** Peritoneal lavage fluids of immunized (*n* = 5) or normal mice (*n* = 5) were collected, stained, and observed with a microscope at 12 h after being infected with 10^5^ CFU WT. Arrows indicate the bacteria. **C, D** Number of bacteria in peritoneal washes, liver, spleen, lung, and blood of immunized or normal mice were determined at 12 h after infection with 10^5^ CFU WT. **E** Survival curves of immunized or normal mice challenged with 10^4^ or 10^5^ CFU WT (*n* = 7 for each PBS group and *n* = 8 for each Δ*kbvR* immunized group) (*P* < 0.0001).

The attenuated virulence and stimulated antibody production of *kbvR* mutant strain made it a candidate for consideration as a live attenuated vaccine. Thus, the ability of Δ*kbvR* strain to confer protection against lethal intraperitoneal challenge with hypervirulent *K. pneumoniae* strain NTUH-K2044 (WT, LD_50_ = 500 CFU) was tested. BALB/c mice were subcutaneously immunized with 10^5^ CFU Δ*kbvR* or PBS control at two doses, 14 days apart. A total of 2 weeks after the second vaccination, mice were intraperitoneally challenged with 10^4^ or 10^5^ CFU WT. At 12 h after the challenge, 5 mice of immunized or normal control mice infected with 10^5^ CFU WT were sacrificed. The peritoneal washes were smeared, stained, and observed with a microscope. No bacteria were observed in the peritoneal lavage fluids of immunized mice. However, plenty of *K. pneumoniae* were found in the abdominal cavities of control mice (Figure 4A and B). The blood, peritoneal washes, liver, lungs, and spleen were harvested to calculate the bacterial number. Immunized mice exhibited significant reductions in organ colonization in the peritoneal washes (1.86-log reduction), liver (1.9-log reduction), spleen (1.6-log reduction), lungs (1.74-log reduction), and blood (3.45-log reduction) compared with the bacterial number of PBS controls (Figure 4C and D). Three of five immunized mice exhibited no detectable bacterial colonization in the blood. By 2 weeks post-challenge, none of the control mice survived beyond 48 h. By contrast, the immunized group had survival rates of 100% and 87.5% for 10^4^ CFU and 10^5^ CFU challenge, respectively (Figure 4E). Overall, these results suggest that the immunization with 10^5^ CFU Δ*kbvR* conferred significant protection in mice against *K. pneumoniae* challenge.

### Antisera from immunized mice increase uptake of *K*. *pneumoniae* by macrophages in vitro

**Figure 5:**
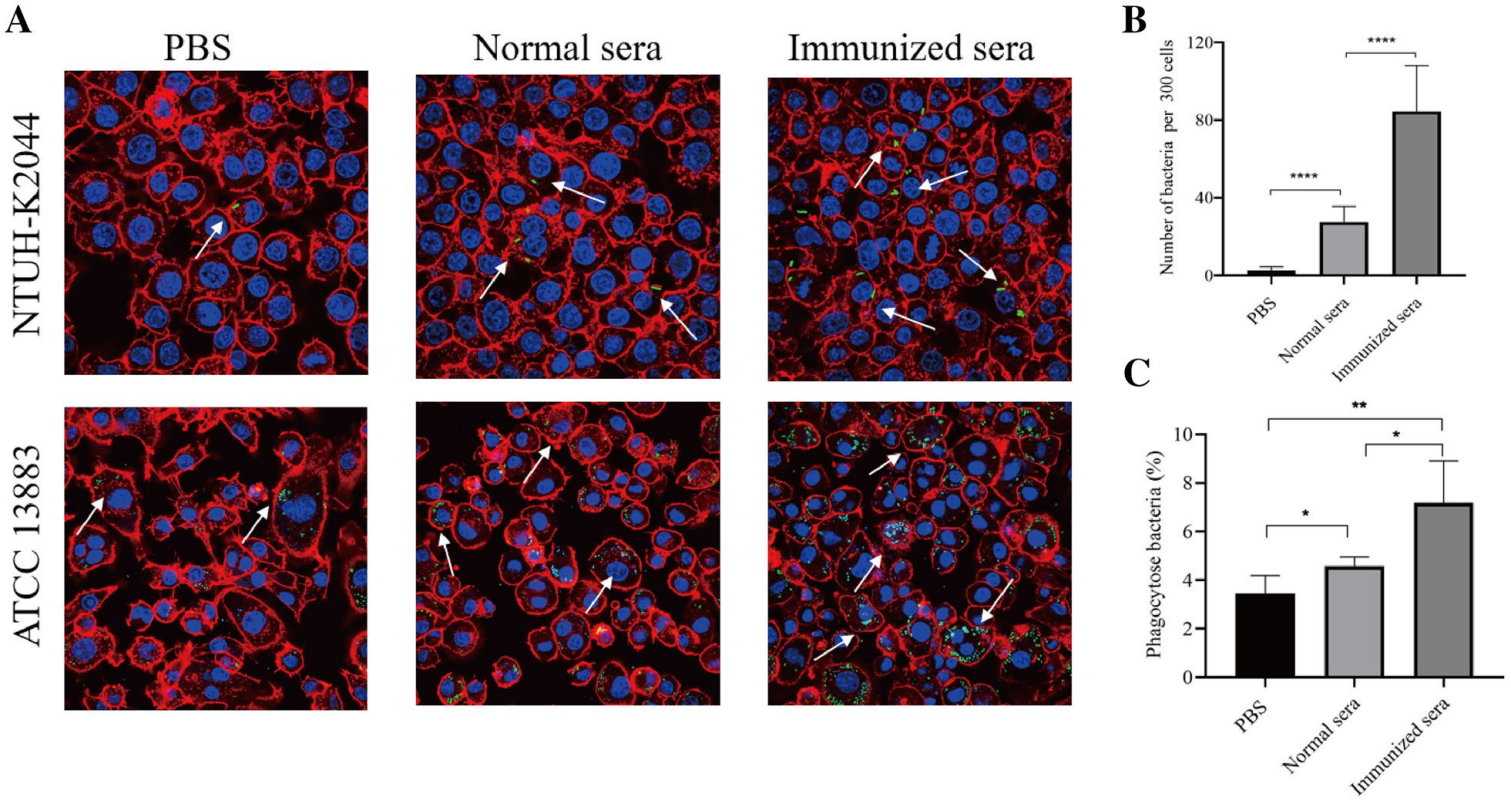
**Determination of opsonophagocytic activity with immunized sera against NTUH-K2044 and ATCC 13,883 by confocal micrograph. A** RAW264.7 macrophage cells were infected with the serotype K1 NTUH-K2044 or the serotype K3 ATCC 13,883 containing the plasmid pLac-EGFP (green) at an MOI of 50 with or without 1:1100 final dilutions of sera from PBS-inoculated normal mice or 10^5^ CFU Δ*kbvR*-immunized mice for 120 min, followed by treatment with 500 µg/mL gentamicin and staining with Alexa Fluor 594 dye (red) for actin cytoskeleton and DAPI (blue) for host cell nuclei. The opsonophagocytic activities of sera were observed under a confocal microscope. Arrows denote the intracellular bacteria. Scale bar = 5 μm. **B** The opsonophagocytic activities of sera against NTUH-K2044 were counted under a confocal microscope. Data are presented as the intracellular bacterial numbers in 300 cells from three independent trials. **C** The opsonophagocytic activities of sera against ATCC 13,883 were determined by the phagocytic rate. The mean opsonophagocytic ratio was calculated by the formula = 100 ⋅ CFU of intracellular phagocytized bacteria/CFU of input bacteria. *: *P* < 0.05; **: *P* < 0.01; ****: *P* < 0.0001.

We measured the ability of the antibodies in immunized sera to promote *K. pneumoniae* uptake by opsonophagocytic assays for determining their functional activity. Phagocytic assay revealed that NTUH-K2044 was rarely presented inside macrophages without adding sera (Figure 5A). Although the addition of normal sera from PBS control mice increased the phagocytic activity of macrophages, the pooled sera from Δ*kbvR* immunized mice show statistically significant enhancement in opsonophagocytic activity compared with the pooled normal sera, whose anti-*Klebsiella* titers were 1:3200 and 1:400 respectively. The average opsonophagocytized NTUH-K2044 were about 80 and 30 within 300 macrophages respectively (*P* < 0.0001) (Figure 5B). The opsonophagocytic assay of the sera against the *K. pneumoniae* serotype K3 strain ATCC 13,883 was also tested to evaluate whether the sera antibodies generated by immunization with the serotype K1 NTUH-K2044 Δ*kbvR* cross-reacted with the other *K. pneumoniae* serotype. The anti-phagocytosis ability of ATCC 13,883 against macrophage was much lower than that of NTUH-K2044 (Figure 5A), which may be partly due to the difference in CPS. Meanwhile, the pooled sera from mice immunized with two doses of serotype K1 Δ*kbvR* strain were also able to significantly increase macrophage uptake of ATCC 13,883 over the normal sera from PBS immunized animals (7.175% versus 4.575%, *P* < 0.05) (Figure 5C). The antisera provided the opsonophagocytic activity not only to the K1 serotype but also to the K3 serotype strain, which implied that immunization with the *kbvR* gene deletion strain of K1 serotype might confer cross-protective immunity against other serotypes of *K. pneumoniae*.

### Antisera from immunized mice increase complement-mediated lysis of *K*. *pneumoniae*

**Figure 6:**
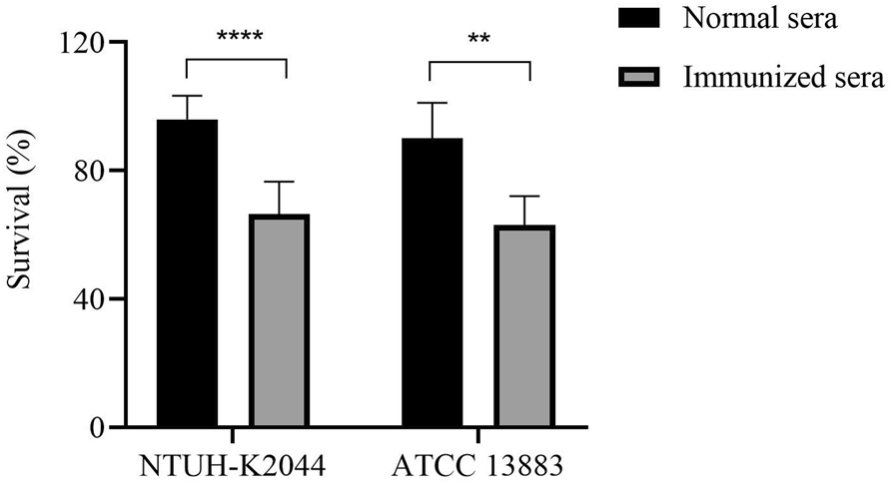
**Immunized sera reduce survival rate of complement-mediated antibacterial test against** ***K. pneumoniae***. The *K. pneumoniae* strain NTUH-K2044 or ATCC 13,883 (10^6^ CFU) were co-incubated with human complement with or without 10% heat-inactivated sera from pooled immunized or normal mice for 2 h at 37 °C. The survival ratio was determined using the formula = 100 ⋅ CFU of experimental wells with mice sera/CFU of control wells without mice sera. **: *P* < 0.01; ****: *P* < 0.0001.

Complement activation is an important component of the innate immunity of the antibacterial immunity. We characterized the complement activation by mice immunized sera in vitro to test whether antisera from immunized mice can increase complement-mediated lysis of *K. pneumoniae*. The *K. pneumoniae* NTUH-K2044 or ATCC 13,883 strain was incubated with the heat-inactivated immunized or normal mice sera, along with human sera as a complement. Compared to the lysis induced by normal sera, the survival rate induced by the immunized sera against NTUH-K2044 and ATCC 13,883 were both significantly decreased (66.4% versus 95.9%; and 63% versus 90%) (Figure 6). The above-mentioned results suggest strong complement activation after immunization with Δ*kbvR*, which might contribute to improving the antibacterial ability of the host immune system.

### Passive transfer of antisera from immunized mice provides protection against *K*. *pneumoniae* infection

**Figure 7:**
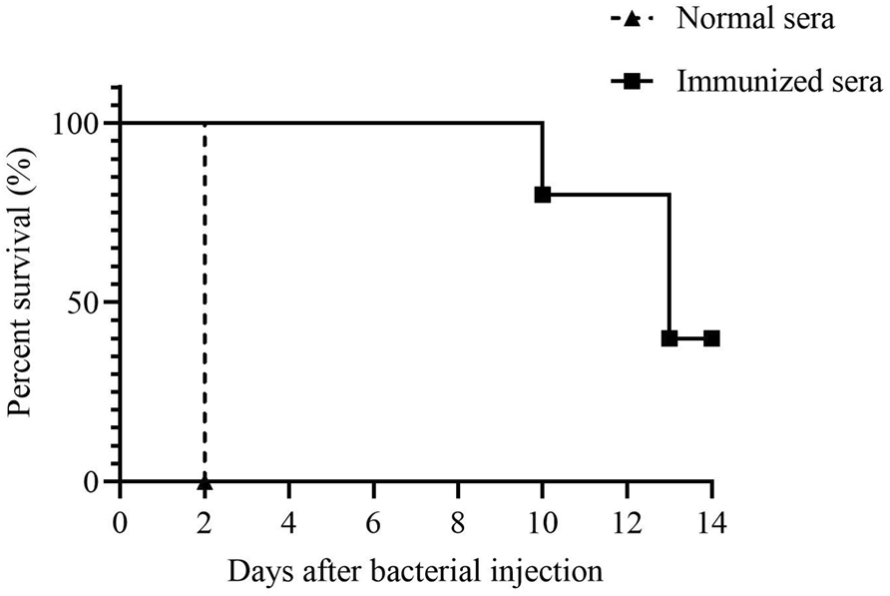
**Protective effect of passive transfer of mice sera against lethal** ***K. pneumoniae*** **infection.** The survival rates of BALB/c mice (*n* = 5/group) injected intraperitoneally with the mixture of 10^4^ CFU WT and immunized or normal mice sera were determined for 14 days (*P* < 0.01).

We determined whether passive transfer of immunized sera to naïve mice could provide protection against infection of *K. pneumoniae* to examine the protective role of antibodies in vivo. Pooled sera (*n* = 5) were obtained from BALB/c mice immunized with 10^5^ CFU Δ*kbvR* (immunized sera) or PBS control (normal sera). The 1 µL pooled immunized or normal sera were intraperitoneally injected into naïve mice (*n* = 5 per group) together with 100 µL *K. pneumoniae* WT (approximately 10^4^ CFU). All mice that were passively transferred with normal sera succumbed to infection within 2 days after injection. On the contrary, all mice treated with immunized sera resolved the infection for 8 days and 40% mice survived for 14 days (Figure 7). These results support the idea that passive transfer of immunized sera is protective against *K. pneumoniae* lethal infection in mice.

## Discussion

*Klebsiella pneumoniae* is an emerging zoonotic pathogen that causes various severe infections in humans and animals, and these infections become untreatable due to the emergence of MDR strains [11, 38]. Therefore, vaccines against *K. pneumoniae* that prevent infection in veterinary practices and humans need to be developed urgently. Among the efforts, live attenuated vaccines are still an appealing approach for animals because of their high immunogenic potential, a greater number of different immunogens, and ability to elicit long-lasting protection. The basic requirements for live vaccines are to reduce the virulence and ensure they contain the highest number of antigenic determinants necessary for inducing protective immunity [39]. In *K. pneumoniae*, the CPS, fimbriae antigens (type I fimbriae and type III fimbriae), and outer membrane proteins (OmpK36 and OmpA) were proven to be potential antigenic determinants for the development of subunit vaccines [17]. Our previous study found that KbvR was associated with the virulence and the synthesis of CPS in *K. pneumoniae* NTUH-K2044 [28]. The decreased expression of CPS in NTUH-K2044 Δ*kbvR* contributes to the attenuation of strain, and the maintained CPS on the surface of Δ*kbvR* strain should still stimulate the protective immune response against the infection of serotype K1 strain. Except for CPS, the synthesis of type I fimbriae was found to be negatively regulated by KbvR and the expression level of type I fimbriae was increased in the Δ*kbvR* strain. Therefore, the weakened physical shielding effect of CPS on the fimbriae along with the reduction in CPS, and the increased synthesis of type I fimbriae on the surface, ultimately enhanced the activity and the chance of type I fimbriae to contact with the immunity system. The highly conserved fimbriae components have been generally shown to be promising candidates for inclusion in vaccines [40–42]. In addition, the structural proteins of type 1 fimbriae have been confirmed as effective protein carriers and immunogens in conjugates [43, 44]. Overall, the maintained CPS and the increased synthesis of type 1 fimbriae made Δ*kbvR* strain a promising live attenuated vaccine that can provide broad antigen exposure and serve as intrinsic adjuvant properties to prime durable immune responses.

Given that Δ*kbvR* could be a live attenuated vaccine candidate, we chose to further characterize the safety and protective effect of this strain using the murine model by subcutaneous route, a used delivery method for live attenuated vaccine. The findings indicated a positive effect of Δ*kbvR* on antibody induction and that the immunization of the Δ*kbvR* strain can stimulate the dose-dependent humoral immune response. The 10^7^ CFU dose-immunized group had higher antibody concentration than the 10^5^ CFU dose-immunized group. Although the mice immunized by 10^5^ CFU Δ*kbvR* strain could provide 100% protection against the 10^4^ CFU WT strain by intraperitoneal challenge, one mouse died among 10^5^ CFU-challenged mice. When the higher dose 10^7^ CFU was used for immunization, 100% protection against 10^5^ CFU challenge at 14 days or even at 42 days after the second immunization were provided (data not shown). However, mice initially inoculated with 10^7^ CFU Δ*kbvR* strain had significantly lower weights and had a local skin side effect. Therefore, the optimum immune dose of Δ*kbvR* with the best protective effect and safety will be fully evaluated in the future.

*Klebsiella pneumoniae* especially K1/K2 serotype strains resist to phagocytosis and complement-mediated killing through the thick capsule at the cell surface [45]. The serum from patients with recurrent *K. pneumoniae* liver abscess increased the opsonizing and killing efficacy, suggesting that the humoral immune response plays an important role in eliminating bacterial infection [46, 47]. In this study, the opsonophagocytic assay and in vitro serum bactericidal activity demonstrated that the mice antisera from 10^5^ CFU Δ*kbvR*-immunized group prompted efficient phagocytosis by macrophages and induced complement-mediated lysis not only against the homologous (K1) serotype strain but also against the heterologous (K3) serotype strain. Thus, the broad immunogens of the live NTUH-K2044 Δ*kbvR* strain might provide a cross humoral protection to other serotype strains. The passive transfer of antisera from Δ*kbvR* immunized mice to naïve mice provided protection against *K. pneumoniae* infection in vivo. The immunization with Δ*kbvR* strain at 10^5^ CFU dose induced an effective antibody protection against infection.

In conclusion, we evaluated the safety and protective effect of KbvR regulator mutant strain. The results suggest that the Δ*kbvR* strain can provide protection against the same serotype and different serotypes, which implies its potential as a live attenuated vaccine against *K. pneumoniae* infection in animals or humans. In the future, the selection of an appropriate immunization dose of the Δ*kbvR* strain which balances between attenuation and immunogenicity, and the cross-protective effect of Δ*kbvR* against additional *K. pneumoniae* serotype strains and MDR strains should be studied subsequently to provide a theoretical basis for developing a commercial vaccine.
